# 

**Published:** 1916-11-18

**Authors:** 


### Sheffield Royal Hospital.

### A HANDY APPLIANCE.

Together with a sample of John Bond's Crystal Palace marking-ink, the excellence of which has secured for it a place in most public institutions, we have received from this firm a clever and handy appliance for stretching linen when in the process of marking; this takes the form of a circular pad, round which is a removable wooden ring. When these are in use a perfect surface for writing is provided, and there is no possibility of the fabric moving during the work.

### PASSING EVENTS AND TOPICS.

### Edith Cavell Homes.

In an autograph letter H.M. Queen Alexandra, when graciously accepting the patronage of the Edith Cavell Homes of Rest for Nurses, expresses the belief that "our sailors and soldiers will be gratified at knowing that an opportunity has been taken of showing national recognition of the labour of the nurses who have broken down in their efforts to alleviate the sufferings of the sick and wounded in the war."

### Dinner to Mr. W. Clive Bridgeman, M.P.

Under the auspices of the Incorporated Society of Hospital Officers a complimentary dinner to Mr. W. Clive Bridgeman was held at the Trocadero Restaurant on Wednesday, the 15th instant. Viscount Knutsford occupied the chair, and was supported by Mr. F. A. Hocking, the President of the Association. The event was organised to express the appreciation of hospital workers of the part played by Mr. Bridgeman in conjunction with those who secured the recent Government concession in respect of relief to hospitals of duty paid upon spirit for medicinal purposes.

### The League of Mercy and Cinemas.

The League of Mercy have decided to accept no contributions that are the result of Sunday performances in cinematograph halls.

### Wounded Allies Relief Committee.

The Wounded Allies Relief Committee, Sardinia House, Kingsway, W.C., have removed to 8 Grosvenor Gardens, S.W., kindly lent to the committee by Lord Ilchester.

### Address to the Lord Mayor.

The new Lord Mayor of London, Sir William Henry Dunn, who is honorary treasurer of Lord Mayor Treloar Cripples' Hospital and College, Alton, was presented at the Guildhall on Lord Mayor's Day with an address signed by the entire staff of the institution. The Lord Mayor was sheriff when the institution was founded in 1907, and has always taken a deep interest in its progress and success, as well as in the welfare of the staff. Before presentation the address was read by the Organising Secretary, Mr. Harper.

### Gifts to Hospitals.

TOWARDS the additions to and equipment of Morton Banks War Hospital a sum of £500 has been given by Mr. Walter Morrison, of Malham Tarn.

IN memory of his two sons, Captain H. H. Bolton and Lieutenant J. Bolton, who were killed in Gallipoli, Mr. H. H. Bolton, of Newchurch in Rossendale, has given £1,000 to Accrington Hospital.

MRS. CATHERINE GRACE DOHERTY WATERHOUSE, of 6 The Esplanade, Scarborough, and formerly of Elland, whose will has been proved, left £100 each to the Halifax Royal Infirmary and the Scarborough Hospital.

HAMPSTEAD GARDEN SUBURB MILITARY HOSPITAL and the Golder's Green Convalescent Home for Wounded Soldiers benefit to the extent of £300 as a result of a gala organised by the Golder's Green Traders' Association.

THE Coventry and Warwickshire Hospital has received a donation of £1,000 from Mr. and Mrs. Percy Loveitt for the endowment of a bed in memory of their only son, Lieutenant Alan Loveitt, who was recently killed in action.

GLASGOW charities benefit by £56,000 under the will of Mr. William P. Lowrie, wine and spirit broker. The Royal Infirmary and Western Infirmary will each receive £10,000, and the Victoria Infirmary and Royal Sick Children's Hospital each £3,000.

THE Glamorgan branch of the British Red Cross Society announced several interesting gifts last week. Two Penarth gentlemen, Messrs. S. Hansen and Frank Shearman, have each presented motor-ambulances for conveying the wounded from Cardiff and Whitchurch stations. A recreation-room is to be added to Penarth Red Cross Hospital at the expense of Mr. A. Mitchelson, and Mr. J. Palin Evans has given 100 guineas to furnish it.

UNDER the will of the late Major W. P. Bennett, R.G.A., of Fleet, Hants, who was killed in action on July 15, the following charities benefit to the extent of £250 each : Dr. Barnardo's Homes, Marlborough College Mission, the Hospital for Sick Children, Great Ormond Street, the National Homes for Homeless and Destitute Children, the Royal Artillery Charities, the Poor Clergy Relief Corporation, the School for Daughters of Officers, and the London Hospital.

### Matrons' and Sisters' Appointments.

GUILDFORD, ROYAL SURREY COUNTY HOSPITAL.—Miss M. Carlin has been appointed sister. She was trained at Sheffield Royal Hospital, where she was later sister of the out-patient department, and has since been sister at Stroud General Hospital, at the East Sussex Hospital, Ipswich, and at the Auxiliary Military Hospital, Griffithstown, near Newport.

BROMBOROUGH AUXILIARY MILITARY HOSPITAL.—Miss V. Cullum has been appointed sister. She was trained at the Royal Devon and Exeter Hospital, Exeter, and has since been sister at the Western Hospital, Torquay.

**NOTE.—Particulars of Appointments for insertion in this column will be welcomed.**

### The Book Market.

LIST OF NEW BOOKS RECEIVED FROM THE FOLLOWING PUBLISHERS:—

Geo. Allen and Unwin, Ltd. : "My Experiences on Three Fronts." By Sister Martin-Nicholson. 4s. 6d. net. "The Flogging Craze." By Henry S. Salt. 2s. 6d. net.

H. K. Lewis and Co., Ltd. : "The Biology of Tumours." By C. Mansell Moullin. 2s. 6d. net.

Cassell and Co. : "Clinical Methods." By Robert Hutchison, M.D., F.R.C.P., and Harry Rainy, M.D., etc. 10s. net. "Eighteen Months in the War Zone." By Kate John Finzi. 6s. net.

T. Fisher Unwin, Ltd. : "With the Russian Wounded." By Tatiana Alexinsky. 2s. 6d. net.

A. and F. Denny : "Dreams, what they Are and what they Mean." By J. W. Wickwor. 1s. net.

Hodder and Stoughton : "The Red Cross in France." By Granville Barker. 2s. 6d. net.

Wm. Heinemann : "In German Hands." By Charles Hennebois. 3s. 6d. net.

Lippincott and Co. : "Care and Feeding of Infants and Children." By W. R. Ramsey, M.D. 9s. net.

Rates of Subscription: Twelve months. 6/6; Six months, 4/-; Three months, 2/-; post free to any address in the United Kingdom. Abroad, Twelve months, 8/8: Six months, 5/-; Three months, 2/6, post free. The Hospital "Index" to Volume LX., April to September 1916, is now ready, price 2½d. per copy, post free. Address The Manager, "The Hospital," The Hospital Building, 28/29 Southampton Street, Strand, London, W.C.

